# Directed Motor-Auditory EEG Connectivity Is Modulated by Music Tempo

**DOI:** 10.3389/fnhum.2017.00502

**Published:** 2017-10-18

**Authors:** Nicoletta Nicolaou, Asad Malik, Ian Daly, James Weaver, Faustina Hwang, Alexis Kirke, Etienne B. Roesch, Duncan Williams, Eduardo R. Miranda, Slawomir J. Nasuto

**Affiliations:** ^1^Brain Embodiment Laboratory, Biomedical Engineering Section, School of Biological Sciences, University of Reading, Reading, United Kingdom; ^2^Department of Electrical and Electronic Engineering, Imperial College London, London, United Kingdom; ^3^School of Psychology, University of Reading, Reading, United Kingdom; ^4^Centre for Integrative Neuroscience and Neurodynamics, University of Reading, Reading, United Kingdom; ^5^Brain-Computer Interfacing and Neural Engineering Laboratory, Department of Computer Science and Electronic Engineering, University of Essex, Colchester, United Kingdom; ^6^Interdisciplinary Centre for Computer Music Research, University of Plymouth, Plymouth, United Kingdom

**Keywords:** coherence analysis, imaginary coherency, electroencephalography (EEG), music tempo, brain connectivity analysis

## Abstract

Beat perception is fundamental to how we experience music, and yet the mechanism behind this spontaneous building of the internal beat representation is largely unknown. Existing findings support links between the tempo (speed) of the beat and enhancement of electroencephalogram (EEG) activity at tempo-related frequencies, but there are no studies looking at how tempo may affect the underlying long-range interactions between EEG activity at different electrodes. The present study investigates these long-range interactions using EEG activity recorded from 21 volunteers listening to music stimuli played at 4 different tempi (50, 100, 150 and 200 beats per minute). The music stimuli consisted of piano excerpts designed to convey the emotion of “peacefulness”. Noise stimuli with an identical acoustic content to the music excerpts were also presented for comparison purposes. The brain activity interactions were characterized with the imaginary part of coherence (iCOH) in the frequency range 1.5–18 Hz (δ, θ, α and lower β) between all pairs of EEG electrodes for the four tempi and the music/noise conditions, as well as a baseline resting state (RS) condition obtained at the start of the experimental task. Our findings can be summarized as follows: (a) there was an ongoing long-range interaction in the RS engaging fronto-posterior areas; (b) this interaction was maintained in both music and noise, but its strength and directionality were modulated as a result of acoustic stimulation; (c) the topological patterns of iCOH were similar for music, noise and RS, however statistically significant differences in strength and direction of iCOH were identified; and (d) tempo had an effect on the direction and strength of motor-auditory interactions. Our findings are in line with existing literature and illustrate a part of the mechanism by which musical stimuli with different tempi can entrain changes in cortical activity.

## Introduction

Our ability to spontaneously perceive the beat of a musical piece—that is, the most prominent accent, periodicity or rhythmic unit (meter) in the piece—is fundamental to the experience of music and is independent of prior musical training (Large et al., 2002). The mechanism behind this spontaneous building of the internal beat representation from music is largely unknown. The resonance theory for beat perception proposes that the perception of periodicities in music is accomplished through an internal mechanism underlined by entrainment of neural populations that resonate to the frequency of the beat (Large and Kolen, 1994; Large, 2008) and studies by Nozaradan et al. (2011, 2012) show that this effect is measurable in electroencephalogram (EEG) activity. Such beat-related changes in the EEG are characterized by EEG entrainment, the phenomenon that oscillations in the EEG increase their activity when their frequency corresponds to the musical beat (dominant tempo). Nozaradan et al. (2015, 2017) report significant periodic increases of EEG amplitude in the time domain at the same frequency as the beat, and multiple peaks at frequencies corresponding to the envelope of the rhythmic pattern in the frequency domain (i.e., frequencies that are directly related to the beat; Nozaradan, 2014). However, it has been shown that spectral representations of rhythms are sensitive to acoustic features of the tones that make up the rhythm, such as tone duration, without affecting beat perception; thus, frequency-domain representations of rhythms are dissociable from beat perception, and entrainment should be interpreted with caution (Henry et al., 2017).

The majority of studies that look at the effects of tempo (the speed of the beat, usually expressed as beats per minute) on brain activity concentrate on spectral features, with effects mainly identified in the α (Yuan et al., 2009) and β ranges (Hurless et al., 2013) of the neural activity. For example, activity in the β range was found to increase when listening to more activating, i.e., faster paced, music (Hurless et al., 2013). Beta activity (14–25 Hz) in particular is associated with higher arousal, which could explain why it was more prominent in music with faster tempo as opposed to slower, more calming music—in fact β activity increased with increasing tempo (Höller et al., 2012). An additional explanation is that the more upbeat music may influence motor regions due to the relationship between activating music and dancing, which in turn results in increased β activity (Höller et al., 2012). Activity in the α range (8–13 Hz) was also found to be associated with tempo modulations. Daly et al. (2014a) report strong α range event-related desynchronization over the left motor cortex, correlated with the variation in musical tempo. In a related study, Yuan et al. (2009) looked at changes in EEG spectral power when listening to tempo-transformed music. The main finding was a decrease in EEG α power as the tempo moved further from the tempo of the original piece. Two possible explanations for this were: (i) increases in brain resources needed to process perceptual information associated with the transformed tempo; and (ii) higher brain activity related to changes in emotional responses caused by the unnatural and odd tempo-transformed music.

It has also been shown that each individual has a preferred musical tempo, and the preferred tempo for the population peaks slightly above 120 beats per minute (2 Hz). The neural correlates of this preferred tempo have been identified as arising from neural activity in the motor cortex and are significantly correlated with the β rhythm (Bauer et al., 2014). This is not surprising as a strong relationship between movement and beat perception has previously been identified: movement influences the perception and metrical interpretation of musical rhythms (Phillips-Silver and Trainor, 2007; Iversen et al., 2009) and listening to rhythmic sequences activates specific motor areas (Schubotz and von Cramon, 2001; Grahn and Brett, 2007; Zatorre et al., 2007; Chen et al., 2008; Bengtsson et al., 2009; McAuley et al., 2012). Of particular interest is the relationship between the preferred musical tempo with the preferred frequency in locomotion (MacDougall and Moore, 2005) and the identification of the contribution of motor areas to rhythm generation and perception (Janata and Grafton, 2003). However, Nozaradan (2014) has identified dampening of the selective enhancement of the neural response to frequencies that correspond to the beat when the tempo of the musical piece is either too fast or too slow. The nature of this neural modulation also differs across individuals and it can be observed as either increased or decreased excitability as tempo approaches the preferred individual tempo; this difference can be due to factors such as attention, musical experience and rhythmic ability (Grahn and Rowe, 2009; Michaelis et al., 2014). It has been suggested that activity in the β band may play a role in this auditory-motor co-ordination (Fujioka et al., 2009, 2012). These findings seem to suggest the existence of a (long-range) neural coupling mechanism between auditory and motor cortices underlying the neural correlates of rhythm perception and production, which has yet to be studied (for a more detailed review of this sensorimotor synchronization see the review by Repp and Su, 2013).

In this study we extend previous findings to investigate the modulation of neural connectivity patterns while participants listen to musical stimuli that target a “peaceful” affective state, played at 4 different tempi. In our previous work we investigated the neural correlates of emotional responses to a varied set of musical stimuli and identified areas with significant power asymmetry and functional connectivity (i.e., temporal correlations) during different induced emotions (Daly et al., 2014b). These were significant in the higher β (18–22 Hz) and γ (35–39 Hz) bands, and this long-range functional connectivity always involved the pre-frontal cortex. However, the neural relationships arising from listening to musical pieces at different tempi have not been previously investigated. Thus, in the current study we are interested in studying the underlying brain coupling during music listening and how this is affected by changes in tempo in particular. To measure this coupling we use the imaginary part of coherence. Coherence is a widely used measure to infer linear non-directional interactions between different areas, but its main weakness is that it is strongly affected by volume conduction. In contrast, “non-interacting sources cannot explain a nonvanishing imaginary coherence”; therefore, the imaginary part of coherence cannot be generated as an artifact of volume conduction (Nolte et al., 2004). This is important in studies with less than 64 electrodes, such as the current study, where methods that deal with volume conduction are not beneficial. Moreover, the current study is exploratory and it is important to be confident that any significant results identified are a result of some coordinated brain activity that is taking place and not of volume conduction.

## Materials and Methods

### Measurements

#### Participants

Twenty-one volunteers, comprising staff and students from the University of Reading and members of the public, participated in this experiment. They were recruited using University emailing lists and word of mouth. This study was carried out in accordance with the recommendations of the University of Reading Research Ethics Committee guidelines. All subjects gave written informed consent in accordance with the Declaration of Helsinki and were compensated £10 for their time. The protocol was approved by the Head of School, following the recommendation of the University of Reading Research Ethics Committee.

The median age of the participants was 25 (range 21–30, standard deviation 2.65), all were right-handed, and 11 were female. Musical training of the participants was determined using a self-report questionnaire: six participants indicated no training, seven only attended obligatory music classes at school, two attended optional music classes at school, five had private music lessons, and one attended music college.

#### EEG Recording

EEG was recorded via a Brain Products BrainAmp EEG amplifier (Brain Products, Germany). Nineteen channels were positioned according to the international 10/20 system for electrode placement (Fp1, Fp2, F7, F3, Fz, F4, F8, T3, C3, Cz, C4, T4, T5, P3, Pz, P4, T6, O1 and O2), referenced to electrode FCz and a ground electrode at AFz. The EEG data were sampled at 1 kHz. Electrode impedances were kept below 5 kΩ for 17 of the participants and below 15 kΩ for all participants. For three participants, channel O2 was excluded from analysis as it had impedance greater than 100 kΩ due to a fault with the EEG input box.

#### Musical Stimuli

The generation and validation of a set of 56 piano music excerpts that convey four intended emotions (happiness, sadness, threat, and peacefulness) is reported in Vieillard et al. (2008). For our experiment, 12 excerpts were selected from this dataset as base clips by uniformly drawing three excerpts without replacement from each emotional category. Using the MIDI files of these base clips, a 50 beats per minute (bpm), 100 bpm, 150 bpm and 200 bpm MIDI version of each clip was created using Anvil Studio by Willow Software^1^. In this process, care was taken to ensure that all versions of a clip had similar content, mainly by constructing the faster versions by looping the slower ones. Each constructed MIDI file was synthesized to a piano WAVE file. The conversion to a piano instrument was performed with the damped version of the “HS Classic Grand Piano” (SoundFont format), freely available by DSK Music and Hispasonic^2^. Each WAVE file was truncated to have length of 9.5 s; this length was long enough to evoke the desired affect and short enough to allow more trials to be included in each session. The last second was faded out by decreasing the amplitude linearly to 0 dB.

Noise clips were used as a means of controlling neural coupling mechanisms that arise as a result of brain response to stimulation with identical acoustic content, but which lacks the specific musical structure. For every music clip, a corresponding noise clip was constructed using the un-windowed Fourier transform algorithm for surrogate data generation described in Theiler et al. (1992). Using this method, surrogate data is generated by computing the Fourier transform of a signal, randomizing the phases by multiplying each complex amplitude with e^iφ^, where φ is uniformly drawn from [0, 2π], and then taking the inverse Fourier transform of the resulting construct. Phases were randomized by being randomly permuted between frequencies so that the phase distribution retained its original shape. As with the music clips, the last second of the noise clips was faded out by decreasing the amplitude linearly to 0 dB. Noise clips have the same average power spectra as their corresponding music clips and, thus, the corresponding music and noise clips have the same energy. The generated noise stimuli do not have a specific temporal structure and, thus, no tempo *per se*, but we can refer to the tempo of the original music stimuli from which the noise clips were generated.

In total, participants were presented 96 clips in random order (48 music clips comprising 12 base clips each at 4 tempi and a corresponding noise clip for each music clip).

#### Paradigm

The experiment consisted of six runs of EEG recording, and lasting approximately 50 min. Participants had their eyes open throughout the experiment. Resting state (RS) recordings were made in the first run in which participants were instructed to rest while fixating on a cross in the center of the screen for 5 min. The four intervening runs comprised 24 trials each. Each trial began with a 200 ms beep and a fixation cross in the center of the screen. After a 2 s interval, a 9.5 s clip was played. This was followed by a 1 s pause after which participants were asked to rate the music on a scale of 1–9 in terms of induced valence (unpleasant to pleasant) and arousal (calm to intense) via self-assessment manikins (SAMs). These two questions were presented on the screen simultaneously, and their layout order was randomly switched between trials. Before the experiment, participants were instructed through an information sheet, a slideshow, a practice trial and a reminder by the investigator to report only their induced emotions. Each trial ended with a distraction task in which participants were presented with a selection of different colored shapes, uniformly distributed across the screen, and instructed to click on all the red squares. The distractor task was used to reduce inter-trial bleed over effects between different stimuli and ensure that neighboring trials were less homogeneous (Liu et al., 2015). The inter-trial interval was between 2–5 s. After each run of 24 trials, participants took a short break, during which they were asked if they felt any fatigue. None of the participants reported that they felt fatigued.

### Imaginary Part of Coherence

Coherence is a measure of the linear relationship of two time-series at a particular frequency. It can be thought of as the equivalent of correlation in the frequency domain. Given two time-series, X = [x_1_,x_2_,…,x_T_] and Y = [y_1_,y_2_,…,y_T_], we define the cross-spectrum at frequency *f*,

(1) $$S_{xy}(f)\;=\;\langle X(f)Y^{*}(f)\;\rangle$$

where X(.), Y(.) are the Fourier transforms of the original time series, * denotes complex conjugation and <…> is the expectation over a sufficiently large number of epochs. Coherence can now be defined as the cross-spectrum normalized by the spectra of **X** and **Y**, which are denoted as S_xx_(f) and S_yy_(f) respectively:

(2) $$C_{xy}(f)\;=\;\frac{S_{xy}(f)}{\sqrt{S_{xx}(f)S_{yy}(f)}}$$

Since the cross-spectrum is a complex number then coherence is also, by definition, a complex number. It is, thus, common to use the magnitude of this measure, the magnitude squared coherence (MSC), as a measure of connectivity:

(3) $$MSC_{xy}(f)\;=\text{|}C_{xy}(f)\text{|}$$

MSC is bounded between 0 (no similarity) and 1 (maximum similarity). However, one limitation of MSC is that, just like many other measures of connectivity, it is affected by volume conduction. A number of transforms exist that can reduce the effect of volume conduction, e.g., Laplacian transform, but these work best when more than 64 electrodes are available (Cohen, 2014). In contrast, Nolte et al. (2004) have shown that the imaginary part of coherence, Imag(C_xy_(f)), is not susceptible to such volume conduction artifacts, while also possessing some additional advantages over MSC. The imaginary part of coherence (the term “imaginary coherence”—iCOH—will be used from this point on for simplicity) exploits the fact that the imaginary component represents interactions occurring with certain time lags. In this respect, even though imaginary coherence is a more conservative measure, the interactions it reveals cannot be biased by volume conduction or a common reference that occur with zero time lag (Nolte et al., 2004). The study of coherent activity using the imaginary part of coherence has since then been applied to a number of studies (e.g., Schlögl and Supp, 2006; Guggisberg et al., 2007; Kelly et al., 2015; Borich et al., 2016; Ohki et al., 2016). Compared with magnitude-squared coherence, imaginary coherence has the additional property of antisymmetry. Antisymmetry is useful for inferring the directionality of interaction and can be extracted from the sign of the imaginary coherence. From Nolte et al. (2004): “if the imaginary part of *C_xy_*(*f*) is positive, then *x* and *y* are interacting and *x* is earlier than *y*; indicating that information is flowing from *x* to *y*”, and vice versa for negative values. In the following sections we use the term iCOH to refer to the imaginary part of coherence.

### EEG Analysis

For this study we analyzed the EEG that was recorded while participants listened to the “peacefulness” musical stimuli. The particular stimuli was chosen as it was previously shown that tempo had a significant effect in music stimuli that was associated with a calming effect (Singer et al., 2016), as well as to allow comparison with a study by Wu et al. (2012), who found changes in EEG phase coherence in the α frequency range during listening to Guqin music and noise. A total of 252 9.5-s length segments (3 peaceful stimuli × 4 tempi × 21 participants) were available for analysis for each type of acoustic stimulus (music, noise). In addition, each participant had a 5-min RS recorded before the start of the experiment, which was split into non-overlapping 9.5-s segments (31 segments total) and imaginary coherence estimates of the RS were also obtained. Prior to any analysis the EEG data was *z*-scored (zero mean, standard deviation 1). No other processing was performed (Supplementary Material provides a comparison of iCOH estimated from data processed with Independent Component Analysis).

The spectra and cross-spectra were estimated using the MATLAB function “*cpsd*” with the following parameters: (i) moving Hanning window with duration 2 s (2000 samples) and 90% overlap; and (ii) frequency range of 1.5–18 Hz (in increments of 0.5 Hz). The imaginary part of coherence (Equation 2) was then extracted and the average iCOH for each frequency range and tempo was estimated over all subjects. This was repeated for RS, music stimuli and noise stimuli. The effect of acoustic stimulation was characterized by comparing changes in iCOH “sources” and “sinks” (sum of iCOH flow into, and out of, a specific electrode respectively) for RS, music and noise. The terms “sources” and “sinks” are commonly used in studies of directed connectivity to describe nodes that are mainly drivers or receivers of activity respectively (e.g., Blinowska, 2011). The effect of tempo was characterized by identifying statistically significant interactions between different tempi when contrasting iCOH for music and noise stimuli.

We also performed behavioral data analysis. The music stimuli induced a state of “peacefulness”, which is a calm and pleasant affective state. Calmness is described by arousal (the level of physical response) and pleasantness by valence (the emotional content). Thus, “peaceful” stimuli typically have a slow tempo and target high valence (pleasant) and low arousal (calm). However, changes in tempo are associated with changes in arousal: as the tempo increases, so does the arousal (see for example, Husain et al., 2002). Therefore, we also wanted to verify whether the participants’ rating of a stimulus that targeted “peacefulness” was modulated by tempo. For this purpose we looked at how the arousal and valence reported by the participants in each trial for the music or noise stimuli were modulated as a function of the tempo.

The sources/sinks of activity were visualized using BrainNet Viewer for MATLAB (Xia et al., 2013). BrainNet Viewer was used purely for visualization purposes at the electrode level (no source reconstruction was performed), as also done in other studies (e.g., Crobe et al., 2016).

### Statistical Analysis

Each pair of iCOH(x,y) values was assessed for significance from zero by employing the commonly used method of surrogate statistics, whereby iCOH(x,y) is compared with the iCOH obtained from surrogate data. For each pair of (x,y) signals, we constructed 500 surrogate y_s_ signals using the un-windowed Fourier transform algorithm for surrogate data generation described in Theiler et al. (1992). Surrogate data, y_s_, are generated by computing the Fourier transform of y, randomizing the phases by multiplying each complex amplitude with e^iφ^, where φ is uniformly drawn from [0, 2π], and then taking the inverse Fourier transform of the resulting construct. We generated 500 such surrogate signals, estimated the surrogate iCOH values and obtained the 95th percentile of the resulting surrogate distribution, which we denoted as iCOH(x,y_s_). The original iCOH(x,y) value is then set as follows:

$$iCOH\left(x,y\right)\;=\;\{\begin{array}{cc} iCOH\left(x,y\right), & if\;iCOH\left(x,y\right)\;\geq \;iCOH\left(x,y_{s}\right)\\ 0, & otherwise \end{array}$$

We next performed statistical testing to assess the significance between the mean iCOH for different tempi, at each of the frequency ranges investigated and for the conditions music, noise and the differential (music-noise). For each participant, we averaged the iCOH values within each frequency range. At a group level, we compared the obtained frequency-means for each tempo using a non-parametric Wilcoxon signed rank test (*α* = 0.05), as iCOH distributions were not Gaussian (Kolmogorov-Smirnov test). The significance level (*α* = 0.05) was corrected for multiple comparisons using the Holm-Bonferroni (HB) correction (Shaffer, 1995), a more powerful alternative to the commonly used Bonferroni correction method (Cohen, 2014). To isolate any significant tempo-induced differences between music and noise with respect to spatial location, we used again a Wilcoxon signed rank test (*α* = 0.05, corrected for multiple comparison with HB correction) to test for significant effect of tempo between the differential (*iCOH*_music_ (*x_i_*,*y_j_* − *iCOH*_noise_(*x_i_*,*y_j_*)) (where *x*_i_, *y*_j_: signals from sensors *i* and *j*).

## Results

**Figure 1:**
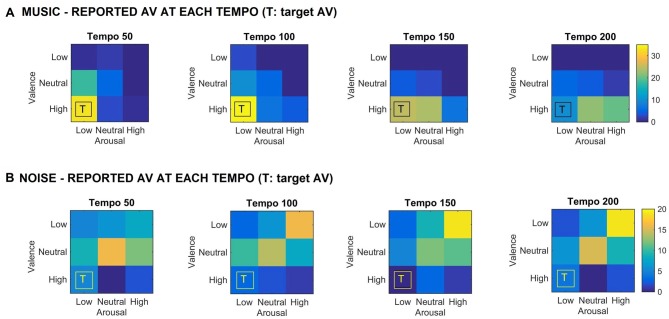
**(A)** Reported arousal-valence (AV) as a function of tempo for the music stimuli. Increase in tempo results in increase of reported arousal. **(B)** Reported AV as a function of tempo for the noise condition. The reported AV appears to converge towards neutral/high arousal and low/neutral valence as tempo increases. “T” indicates the AV that the corresponding music stimulus was targeting. The scale represents the number of trials the participants reported a particular arousal and/or valence for the music stimuli.

**Figure 2:**
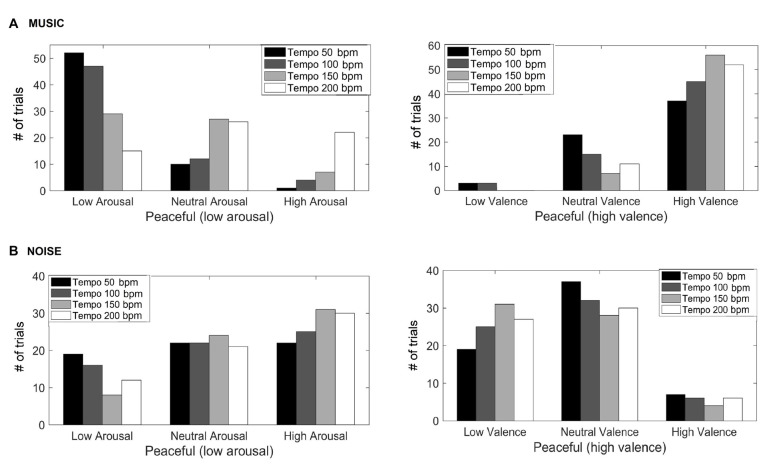
Reported arousal and valence for the music **(A)** and noise **(B)** stimuli. The *y*-axis represents the number of trials the participants reported a particular valence and/or arousal.

Figure 1 shows the valence and arousal reported by the participants as the tempo of the (A) music (top row) and (B) noise (bottom row) stimuli increases respectively. This figure shows the number of trials that a particular combination of arousal-valence (AV) was reported by the participants as a function of tempo. As the tempo increases, the reported AV shifts from the target low arousal-high valence (indicated as “T” on the matrices) to a higher arousal (valence remains high). Figure 2 shows a breakdown into reported arousal and valence, for the (A) music (top row) and (B) noise (bottom row) conditions respectively. Slower tempo is associated with more frequent reports of low arousal. However, the effect of tempo is not as clear-cut for valence, as the majority of participants reported high valence regardless of the tempo. For the corresponding noise stimuli, the majority of participant reports are high arousal and low valence, which are usually associated with “scary” sounds. Tempo does not appear to have a strong effect on these reports, but an association of faster tempo with higher arousal and slower tempo with lower valence can also be seen (Figure 2B).

**Figure 3:**
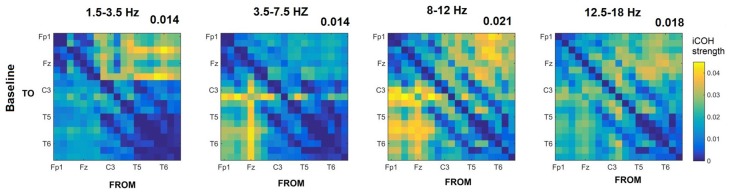
Group mean imaginary coherence (iCOH) for resting state (RS) at different frequency ranges. The corresponding mean iCOH over all connections is also shown in the top right corner of each plot.

**Figure 4:**
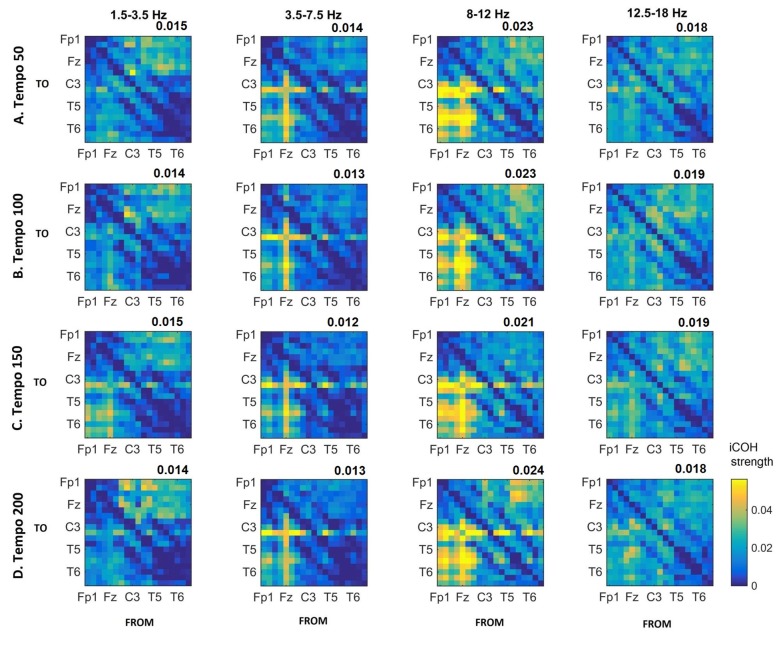
Group mean iCOH for musical stimuli estimated at different tempi (rows) and frequency ranges (columns). The corresponding mean iCOH over all connections is also shown in the top right corner of each plot. Each matrix represents the iCOH for all electrode pairs for the particular tempo at each frequency range. Matrix columns are sources of information flow (“From”), while rows are sinks (“To”). Channel order for each column/row: Fp1, Fp2, F7, F3, Fz, F4, F8, T3, C3, Cz, C4, T4, T5, P3, Pz, P4, T6, O1 and O2.

**Figure 5:**
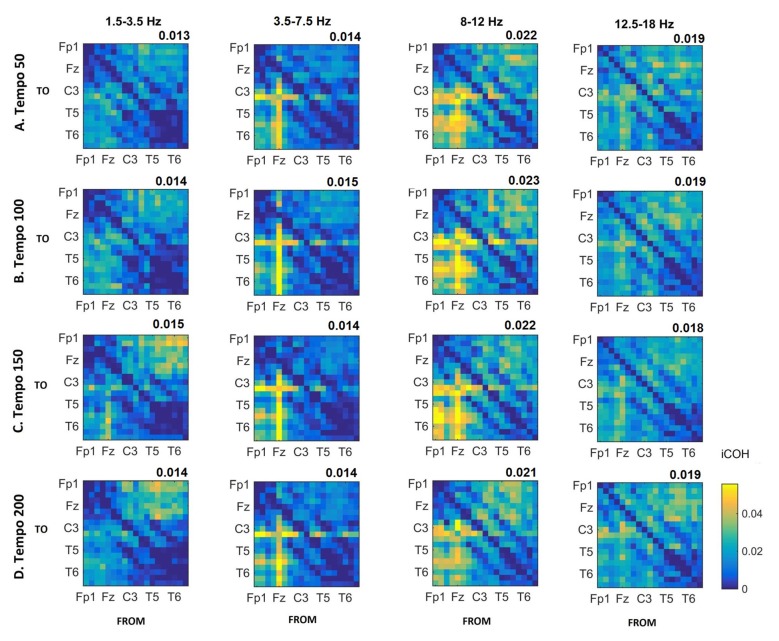
Group mean iCOH for noise stimuli at different tempi (rows) and frequency ranges (columns). The corresponding mean iCOH over all connections is also shown in the top right corner of each plot. Each matrix represents the iCOH for all electrode pairs for the particular tempo at each frequency range. Matrix columns are sources of information flow (“From”), while rows are sinks (“To”). Channel order for each column/row: Fp1, Fp2, F7, F3, Fz, F4, F8, T3, C3, Cz, C4, T4, T5, P3, Pz, P4, T6, O1 and O2.

**Figure 6:**
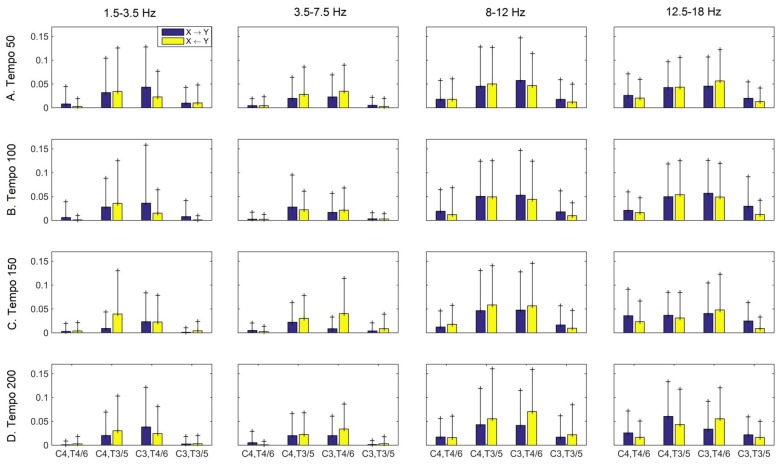
Group mean (and standard deviation) motor (C3, C4) and auditory (T4/6, T3/5) imaginary coherence (iCOH) at different tempi (rows) and frequency ranges (columns). Blue: motor→auditory; Yellow: auditory→motor. We observe increased iCOH in the α and β ranges, and modulation in strength and direction of interaction in different tempi and frequency ranges.

Figures 3–5 show the group average iCOH strength for the RS, music stimuli and noise stimuli respectively, estimated in the frequency range 1.5–18 Hz (see also Supplementary Figure S1 for the corresponding figure for music stimuli obtained from the processed EEG data). The mean iCOH over each frequency range (and tempo for the music and noise conditions) is also provided in Figures 3–5 (top right corner of each plot). Each column represents the sources of interaction (from a specific electrode to all other electrodes) while each row represents the sinks of interaction (to a specific electrode from all other electrodes). The iCOH patterns appear, at a first glance, to be topologically similar for all conditions, with similar levels of mean iCOH strength, which was expected at least for the α range (Wu et al., 2012). Other interactions involving frontal midline locations are also visible in all conditions, most prominent at 3.5–12 Hz in all tempi and conditions. A separate presentation of the motor-auditory connectivity during music is shown in Figure 6. The inter- and intra-hemispheric motor (C3, C4) and auditory (T3, T4, T5, T6) connections are shown for all tempi and frequency ranges. An effect of both frequency and tempo can be seen. There is increased inter-hemispheric iCOH in the α and β ranges, and tempo is found to modulate both the strength and direction of interactions. Supplementary Figure S2 shows the corresponding motor-auditory connections for the processed data; these are very similar to the connectivity obtained from the unprocessed data.

**Figure 7:**
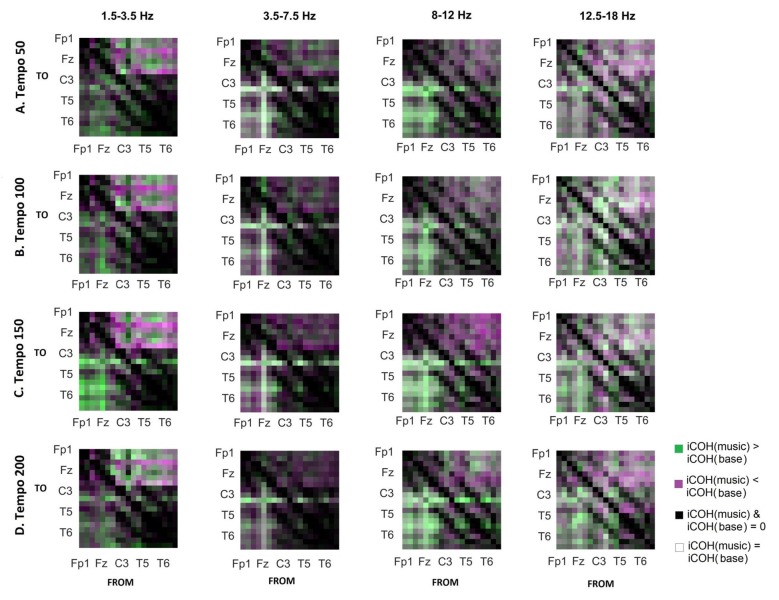
Contrast between RS and music group mean iCOH. Areas in green indicate stronger iCOH for the music condition, while areas in magenta indicate the opposite. Grayscale indicates areas of similar strength; black: areas where iCOH is zero in both conditions; white: areas where iCOH is equal in both conditions.

To isolate the effect of acoustic stimulation we contrasted the RS with music (Figure 7). This type of contrast allows us to identify areas where iCOH is increased (green) or decreased (magenta) due to acoustic stimulation with respect to RS. From this contrast it can be seen that the effect of acoustic stimulation on the strength of iCOH is more isolated to long-range fronto-posterior and fronto-temporal interactions (1.5–12 Hz), while a more spatially broad effect is seen in the β range (12.5–18 Hz).

**Figure 8:**
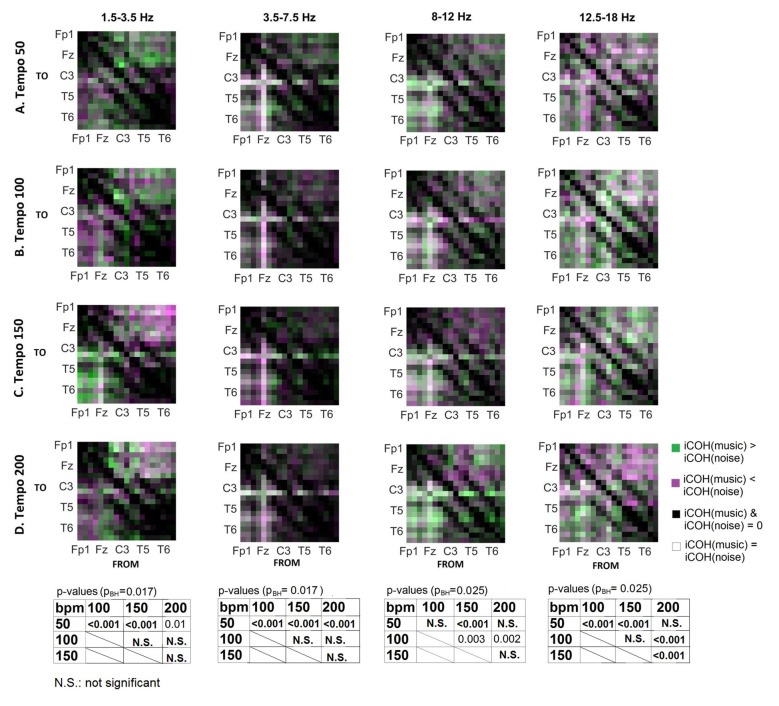
Contrast between music and noise group mean iCOH. Areas in green indicate stronger iCOH for the music condition, while areas in magenta indicate the opposite. Grayscale indicates areas of similar strength; black: areas where iCOH is zero in both conditions; white: areas where iCOH is equal in both conditions. Significant effects of tempo were also identified and corresponding *p*-values are also shown (Wilcoxon signed rank test, *α* = 0.05, corrected for multiple comparisons with Bonferroni-Holm).

To isolate the effect of tempo over and above the effect of acoustic stimulation, we contrasted the group mean iCOH from music and corresponding noise stimuli (Figure 8). This type of contrast allows us to identify areas where iCOH is increased (green) or decreased (magenta) due to the effect of tempo, isolated from acoustic stimulation effects. We examined the statistical significance in iCOH due to tempo at each of the frequency ranges (Wilcoxon signed rank test; *α* = 0.05, *p*_BH_: HB corrected *p*-value). Significant *p*-values between the different tempi are provided at the bottom of Figure 8. Tempo had significant effects in the group mean iCOH strength, which varied across the frequency ranges examined.

**Figure 9:**
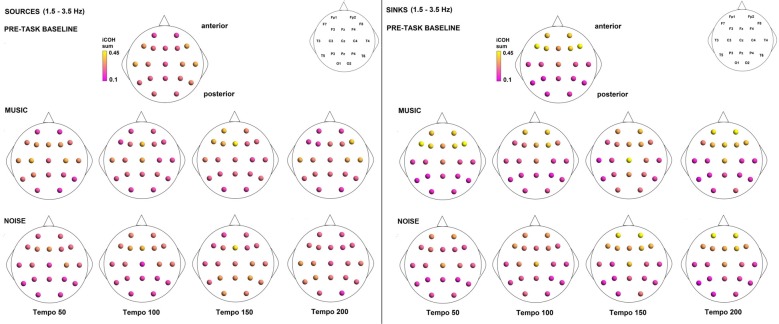
Sources (iCOH flowing outwards) and sinks (iCOH flowing inwards) of activity for RS, music and noise stimuli estimated for the δ (1.5–3.5 Hz) frequency range.

**Figure 10:**
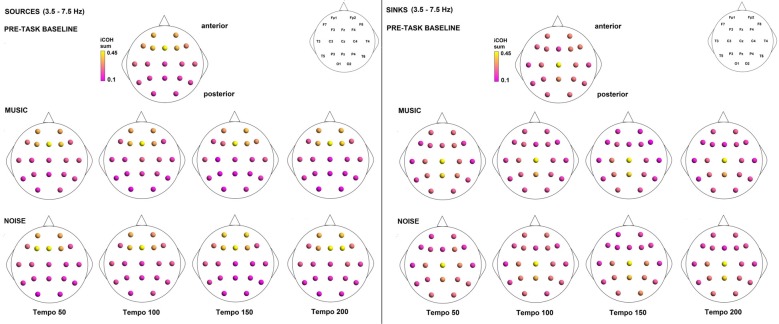
Sources (iCOH flowing outwards) and sinks (iCOH flowing inwards) of activity for RS, music and noise stimuli estimated for the θ (3.5–7.5 Hz) frequency range.

**Figure 11:**
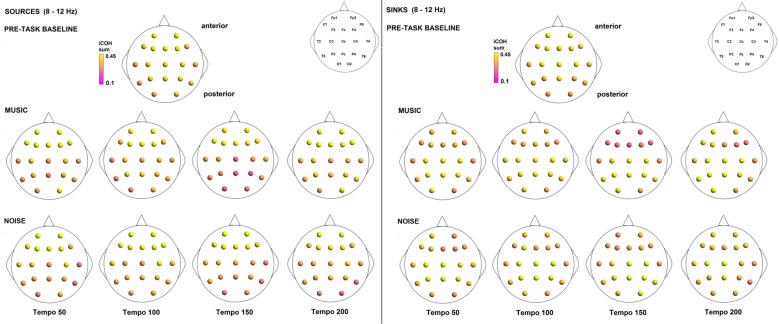
Sources (iCOH flowing outwards) and sinks (iCOH flowing inwards) of activity for RS, music and noise stimuli estimated for the α (8–12 Hz) frequency range.

**Figure 12:**
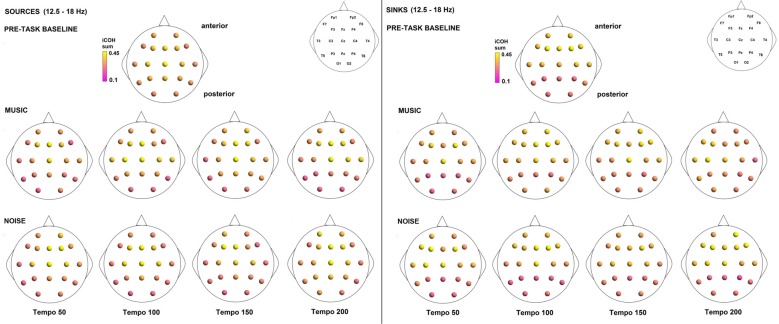
Sources (iCOH flowing outwards) and sinks (iCOH flowing inwards) of activity for RS, music and noise stimuli estimated for the β (12.5–18 Hz) frequency range.

To examine the effect of tempo and acoustic stimulation on the direction of interaction, we estimated the sources (sum of iCOH flowing outwards from an electrode) and sinks (sum of iCOH flowing inwards into an electrode) for RS, music and noise over the four frequency ranges studied (Figures 9–12). To isolate the effect of tempo over and above acoustic stimulation we contrasted the corresponding sources/sinks of music and noise. Tempo had an effect on the distribution of sources and sinks (with the most striking difference observed at 150 bpm, Figure 10), and this implies that tempo affects not only the strength of iCOH, but also the direction of interaction.

## Discussion

As part of this study we investigated a potential mechanism behind the internal beat representation from music. We looked at how tempo may affect the underlying brain interactions represented by imaginary coherence, as a new contribution to the previous literature. We identified changes in both the strength of imaginary coherence as well as topological changes in connectivity as a function of tempo and frequency range.

Our findings can be summarized in the following points: (1) changes in tempo were associated with increased arousal; (2) the RS is characterized by ongoing long-range interactions engaging fronto-posterior areas; (3) music and noise both modulate these interactions, but with a somewhat different effect in the frontal/posterior sources and sinks that can be isolated to significant differences in specific electrode pairs involving temporo-posterior, cortical and frontal locations; and (4) some of the significant differences in music and noise conditions can be isolated as an effect of tempo.

The music stimuli targeted “peacefulness”, which is characterized by high valence and low arousal. Our results revealed that variations in tempo were indeed associated to different subjective levels of arousal, with music played at faster tempo eliciting increased arousal, without much effect on the reported valence. This was in contrast to noise stimuli, where faster tempo was associated with low valence and high arousal, thus the noise stimuli had valence and arousal that were more characteristic of a “scary” emotional state. Thus, participants might tend to rate the music clips “more” pleasant as they are actively comparing them to the noise clips.

We observed long-range interactions at all tempi, with connection strengths that appeared to be modulated by tempo. The iCOH connectivity for RS, music stimuli and noise stimuli (Figures 3, 4) did not differ in terms of topological structure (something that was also seen in Figure 1 of Wu et al., 2012). We observe changes in the strength of the connectivity between the three conditions (Figures 7, 8), as well as in the connectivity strength between motor-auditory connections (Figure 6). The topological similarity is not surprising. Luczak et al.’s (2009) show that, at a population neuronal level, the activity patterns in response to sensory stimuli and spontaneously without sensory input are drawn from a limited “vocabulary”, sampled widely by spontaneous events and more narrowly by sensory responses. It is reasonable to expect that Luczak et al.’s (2009) findings of a population-level limited vocabulary of neuronal patterns would also be somehow reflected in the EEG activity, which is a manifestation of the underlying neuronal activity.

The modulation in the strength of the observed long-range connections in the brain while listening to music may play a role in neural transformation of the rhythm and explain how the information from movement or other sensory modalities contributes to the internal representation of the beat (Nozaradan et al., 2017). This long-range functional connectivity is likely represented in frequencies lower than 30 Hz, while frequencies higher than 30 Hz could reflect a more local scale of neural activity (Nozaradan et al., 2017). Our findings reveal a relationship between motor and auditory cortices in all frequency ranges studied, but most prominent in the α (8–12 Hz) and β (12.5–18 Hz) ranges. These findings are consistent with prior reports in the literature (e.g., Janata and Grafton, 2003; Bauer et al., 2014; Daly et al., 2014a).

Tempo has been shown to affect the ability to synchronize with the beat during finger tapping, with faster tempi being associated with increased accuracy of beat synchronization (Nozaradan et al., 2016). However, this sensorimotor synchronization was found to break down for tempi greater than 2 Hz (120 bpm; Nozaradan et al., 2016), and tempi that are even higher (300 bpm) were found to disrupt the EEG entrainment to the beat-related frequency (Nozaradan et al., 2017). The results reported by Nozaradan (2014), Nozaradan et al. (2015, 2017) were obtained with frequency-tagging and, as argued by Henry et al. (2017), any EEG entrainment identified using this method should be interpreted with caution. On the contrary, iCOH is one of the measures that capture long-range functional interactions and as a result may reveal a more general underlying mechanism of tempo modulation. Schroeder et al. (2010) propose that rhythmic motor oscillations modulate oscillations in the auditory cortex, and auditory perceptual processes may in turn be influenced by them. This is also supported by Phillips-Silver and Trainor (2007) who show that musical rhythm processing is a multisensory interaction relying primarily on reciprocal interactions between auditory-motor systems. Our findings of bidirectional motor-auditory interactions modulated by tempo are in agreement with the literature.

An additional interesting finding is the strong sink of activity at Cz and the strong source from Fz. These strong interactions are most prominent in the θ and α ranges. It is also interesting that these are not unique to the type of stimulus or RS. This is not simply a channel issue as these interactions are not as prominent in all participants or throughout all trials for a single participant. There was no correlation between participant characteristics that could be associated with these interactions, such as sex or musical education. Thus, these interactions are related to the experimental task, either as a direct consequence of auditory stimulation (see Skoe and Kraus, 2010 and references within), activation of premotor cortical areas responsible for movement planning (frontal midline region) as a consequence of an internal timing mechanism linked to rhythm perception (Kaufer and Lewis, 1999; Repp and Su, 2013) or visual stimulation as participants had their eyes open in all conditions and were looking at a fixation cross.

Perhaps the closest study to our own is a study by Wu et al. (2012) investigating the large-scale structure of functional brain networks during music perception using graph theoretical measures obtained from phase coherence networks in the α range (8–13 Hz). Wu et al. (2012) used Guqin music excerpts as musical stimuli, which also has a peaceful effect on listeners, 1/f noise, and silence. Looking at Figure 1 in Wu et al. (2012) there are no striking visible differences between the music, noise and silence conditions. This is in line with our findings in the α range, as well as the additional frequency ranges that we investigated. Wu et al. (2012) report an increase in phase coherence during music perception in contrast to noise and silence, as well as increased connections between prefrontal and frontal regions, and parietal, occipital and temporal regions. This is consistent with our findings in both the statistically significant differences in group mean strength and topology of the iCOH. However, the interactions identified in Wu et al. (2012) are localized. Their particular method of analysis used instantaneous phases, however there was a lack of any pre-processing to remove volume conduction artifacts (64 electrodes). The absence of long-range phase synchrony, coupled with the increased short-range synchrony could be an indicator of volume conduction artifacts.

Imaginary coherence is conceptually easy to estimate, has low computational complexity and is robust to volume conduction artifacts (the imaginary component ignores any relationships at time lag zero; Nolte et al., 2004). The latter is an important advantage, particularly for an exploratory study such as this one: we can be certain that any brain interactions revealed by imaginary coherence result from dependencies that are less likely an artifact of volume conduction. The caveat is, however, that any true interactions at time lag zero or interactions with a time delay that is statistically indistinguishable from zero will not be captured. Methods such as Partial Directed Coherence (PDC) and (direct) Directed Transfer Function (DTF; Blinowska, 2011) are also robust to volume conduction and are more popular methods of choice. However, we must make a distinction between the different types of relationships that these methods capture. Following the definitions by Friston (1994), a measure of functional connectivity estimates temporal dependencies between spatially remote neurophysiological events (e.g., imaginary coherence, Guggisberg et al., 2007), while a measure of effective connectivity explicitly hypothesizes a linear model of dependence (e.g., PDC and DTF). One must, therefore, be clear on what types of dependencies are captured by a particular method. Horwitz emphasizes that the multiple ways by which functional connectivity is determined will not necessarily lead to the same conclusion, and this can even be the case for data from the same task or obtained from the same modality; this is complicated further when one goes from functional to effective connectivity, which is underpinned by a hypothesized model (Horwitz, 2003). The majority of published studies concentrate on a single measure of interaction (for example, Babiloni et al., 2005; Daly et al., 2012; Nicolaou et al., 2012; Wu et al., 2012; Park et al., 2017; Sunwoo et al., 2017) and it is reasonable to expect some differences in the conclusions when different methods are applied, especially if those methods capture different types of functional and/or effective connectivity. Deviations can also be expected if non-linear dependencies are also present and a measure that captures such dependencies is used (imaginary coherence only captures linear dependencies).

We note some limitations of our study. First, the investigations are performed on static coherence networks obtained from the entire EEG segment. This is useful in characterizing generalized changes. However, it may also be interesting to study the changes in connectivity strength at different snapshots throughout the entire stimulus period, as such changes could be important in identifying re-setting of connectivity and modulation of it by the musical stimulus (as per Fujioka et al., 2012) and whether tempo has an effect that is sustained over time—and similarly for the resting period. Second, we have tried here to dissociate the effect of tempo from emotional effects, as supported by literature. Our behavioral findings, however, support a relationship between tempo and arousal. Thus, investigating the connectivity over perceived, rather than intended, affect may reveal additional information related to tempo-related mechanisms of brain activity modulation. Lastly, we were not able to provide a concrete explanation for the strong Fz and Cz connectivity. Even though there are some plausible mechanisms in the literature to explain this, a more thorough investigation must be performed to identify the origins of these connections.

## Conclusion

This study investigated the effect of tempo on brain connectivity during passive listening to musical stimuli designed to induce “peacefulness”, contrasted with passive listening to corresponding noise stimuli generated from the music stimuli at each of the four tempi investigated. Connectivity was measured with imaginary coherence and the estimated networks were analyzed in four frequency ranges spanning 1.5–18 Hz. The current study contributes towards elucidating neural representations of musical beat, although future investigations will need to clarify these mechanisms further, such as looking at tempo irrespective of target and perceived musical affect, and vice versa, i.e., investigating changes related to both tempo and perceived affect, and isolating the nature of the midline interactions. Our findings indicate significant changes to the strength and directionality of long-range functional brain connections, as measured by imaginary coherence, resulting from both acoustic stimulation and variations in tempo. The identified changes in connectivity support an effect of tempo on brain interactions.

## Author Contributions

AM, ID, JW, FH, AK, EBR, DW, ERM and SJN contributed to the conception of the project or design of the study. AM, ID and JW conducted the experiment. NN performed data analysis. NN, AM, ID, FH and SJN contributed to data analysis and interpretation. NN and AM drafting the manuscript. All authors critically revised the manuscript for important intellectual content, approved the manuscript and agreed on publishing it.

## Conflict of Interest Statement

The authors declare that the research was conducted in the absence of any commercial or financial relationships that could be construed as a potential conflict of interest.

## Footnotes

^1^http://www.anvilstudio.com/

^2^https://www.hispasonic.com/

## Supplementary Material

The EEG data analyzed in this paper are openly available from the University of Reading Research Data Archive at http://dx.doi.org/10.17864/1947.124
