# Supplementary material for: Directed Motor-Auditory EEG Connectivity Is Modulated by Music Tempo
Source: Front Hum Neurosci. 2017 Oct 18;11:502. doi: 10.3389/fnhum.2017.00502 (PMC5651276; doi:10.3389/fnhum.2017.00502)
Supplement: Supplementary file 1 [file Data_Sheet_1.pdf]

Supplementary Material

Directed motor-auditory EEG connectivity is modulated by music tempo

Nicoletta Nicolaou[1,6*], Asad Malik[1,4,5], Ian Daly[2], James Weaver[1], Faustina Hwang[1], Alexis Kirke[3], Etienne B. Roesch[4,5], Duncan Williams[3], Eduardo R. Miranda[3], Slawomir J. Nasuto[1]

[1] Brain Embodiment Laboratory, Biomedical Engineering Section, School of Biological Sciences, University of Reading, Reading, UK

[2] Brain-Computer Interfacing and Neural Engineering Laboratory, Department of Computer Science and Electronic Engineering, University of Essex, UK

[3] Interdisciplinary Centre for Computer Music Research, University of Plymouth, Plymouth, UK

[4] School of Psychology, University of Reading, Reading, UK

[5] Centre for Integrative Neuroscience and Neurodynamics, University of Reading, Reading, UK

[6] Department of Electrical and Electronic Engineering, Imperial College London, UK

*** Correspondence:**
Corresponding Author
n.nicolaou@reading.ac.uk

1 Supplementary Figures

The results presented in the manuscript were obtained from the unprocessed EEG. In the supplementary material we show the estimated imaginary coherence (iCOH) for processed data.

First, the EEG was pre-processed using a notch filter (Hamming windowed sinc finite impulse response with automatic filter order estimation as implemented in EEGLab (Delorme and Makeig, 2004)) to remove power line noise between 49 and 51 Hz. Independent component analysis (Infomax ICA (Bell and Sejnowski, 1995), as implemented in EEGLab (Delorme and Makeig, 2004)) was then applied on the EEG of each run to attempt to separate artefacts from components of interest. Each independent component (IC) was visually inspected by authors Daly (6 years of experience) and Malik (1 year of experience), in terms of its time series, scalp map, and power spectrum, and was removed if judged to contain artefacts. Cleaned EEG was re-constructed by inverting the ICA de-mixing matrix and multiplying it by the remaining ICs.

After cleaning via ICA, the EEG of each run was band-pass filtered between 0.5-45 Hz (Hamming windowed sinc finite impulse response with automatic filter order estimation as implemented in EEGLab (Delorme and Makeig, 2004)) to remove high frequency noise components and direct current (DC) drift. Finally, it was visually inspected and marked for electromyographic (EMG) activity, movement, and other artefacts that had not been removed using ICA. Trials that contained any such artefacts or in which the amplitude of the signal exceeded ± 100 μV (the accepted amplitude range of clean EEG (Daly et al., 2012)) were excluded from further analysis.

The processed data was then analyzed following the same procedure described in Section 2.3.

Supplementary Figure 1 shows the group mean imaginary coherence (iCOH) for musical stimuli estimated at different tempi (rows) and frequency ranges (columns) for the processed data. The corresponding mean iCOH over all connections is also shown in the top right corner of each plot. The equivalent figure for the unprocessed data is Figure 4. Supplementary Figure 2 shows the group mean motor (C3, C4) and auditory (T4/6, T3/5) imaginary coherence (iCOH) at different tempi (rows) and frequency ranges (columns) for the processed data. The equivalent figure for the unprocessed data is Figure 6.

Supplementary Figures 1 and 2 show that there are no topological differences in the iCOH for the unprocessed and unprocessed data, but there is a reduction in iCOH strength for the processed data.

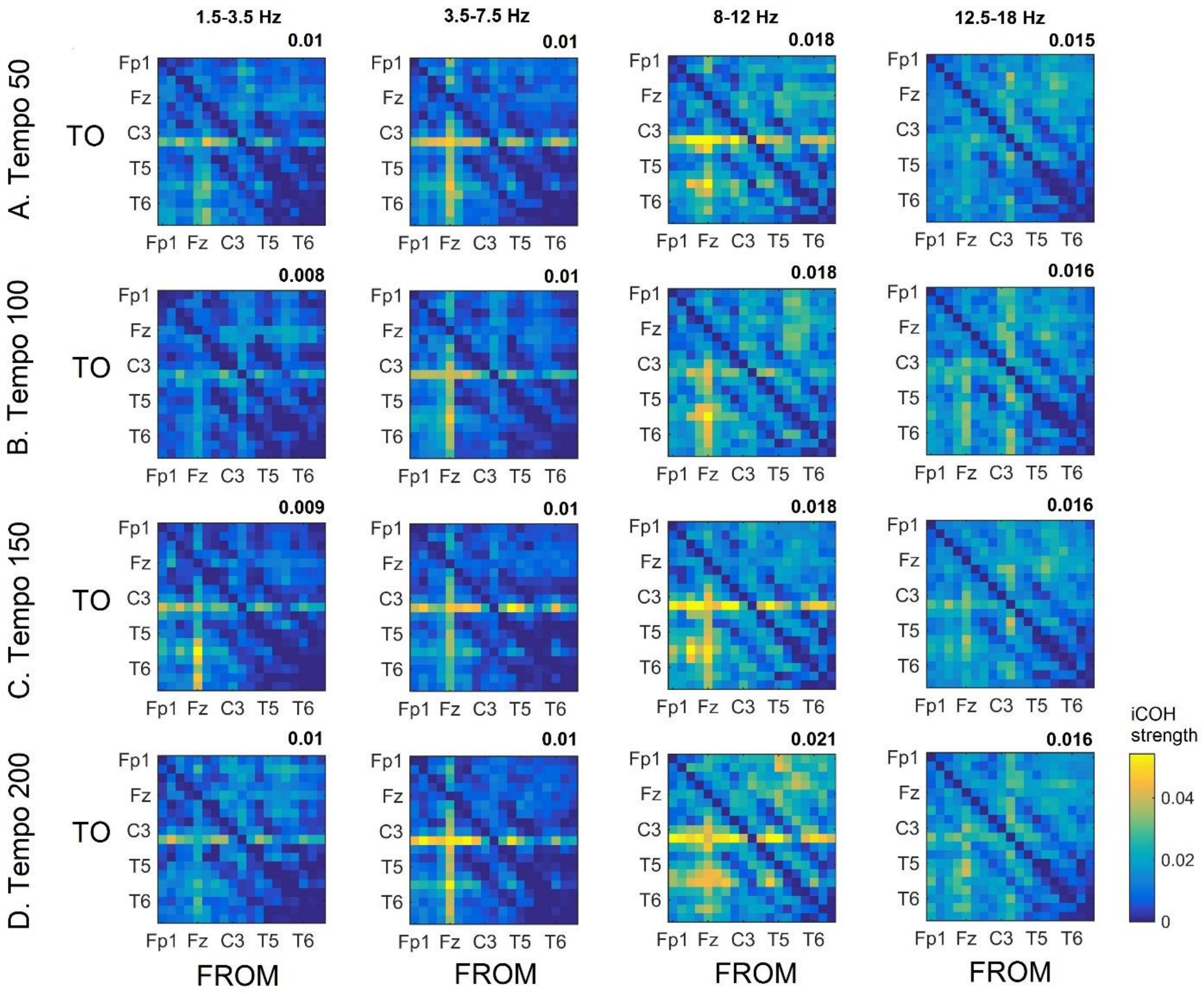

Supplementary Figure 1. Group mean imaginary coherence (iCOH) for musical stimuli estimated at different tempi (rows) and frequency ranges (columns). The corresponding mean iCOH over all connections is also shown in the top right corner of each plot. Each matrix represents the iCOH for all electrode pairs for the particular tempo at each frequency range. Matrix columns are sources of

information flow (‘From’), while rows are sinks (‘To’). Channel order for each column/row: Fp1, Fp2, F7, F3, Fz, F4, F8, T3, C3, Cz, C4, T4, T5, P3, Pz, P4, T6, O1, and O2.

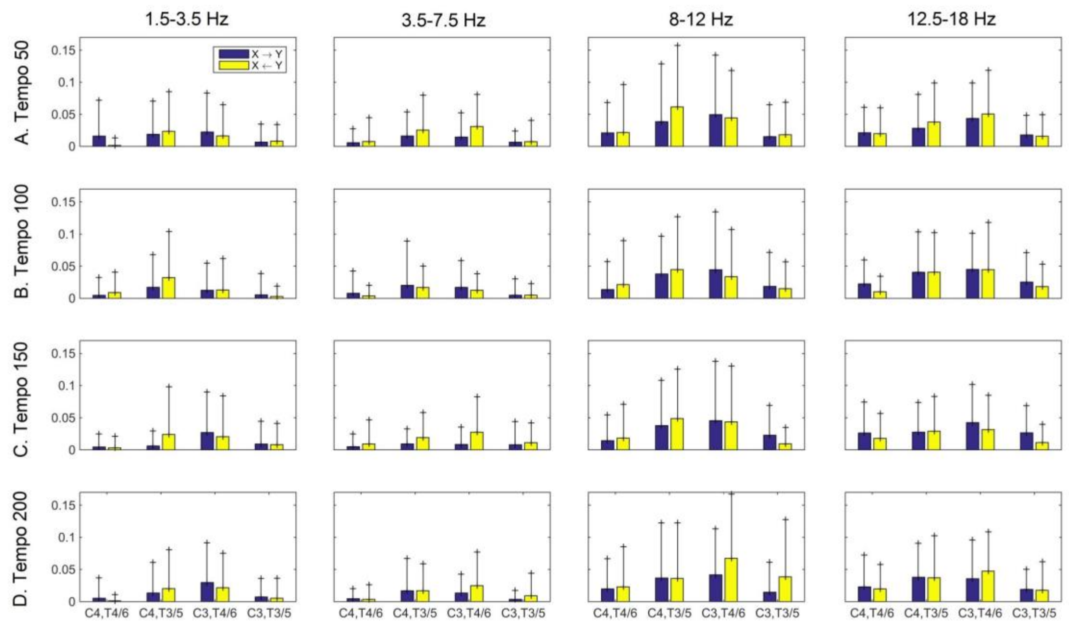

Supplementary Figure 2. Group mean (and standard deviation) motor (C3, C4) and auditory (T4/6, T3/5) imaginary coherence (iCOH) at different tempi (rows) and frequency ranges (columns). Blue: motor→auditory; Yellow: auditory→motor. We observe increased iCOH in the α and β ranges, and modulation in strength and direction of interaction in different tempi and frequency ranges.

References

Bell, A., and Sejnowski, T. (1995). An information-maximization approach to blind separation and blind deconvolution. *Neural Comput* 7(6)**,** 1129-1159.

Daly, I., Pichiorri, F., Faller, J., Kaiser, V., Kreilinger, A., Scherer, R., et al. (2012). "What does clean EEG look like?", in: *34th Annual International Conference of the IEEE EMBS.* (San Diego, CA, USA).

Delorme, A., and Makeig, S. (2004). EEGLab: an open source toolbox for analysis of single-trial EEG dynamics including independent component analysis. *J Neurosci Methods* 134(1)**,** 9-21.
